# The Fungal-Specific Transcription Factor VpFSTF1 Is Required for Virulence in *Valsa pyri*

**DOI:** 10.3389/fmicb.2019.02945

**Published:** 2020-01-10

**Authors:** Alex Machio Kange, Ai Xia, Jierui Si, Bingxin Li, Xiong Zhang, Gan Ai, Feng He, Daolong Dou

**Affiliations:** ^1^Department of Plant Pathology, College of Plant Protection, Nanjing Agricultural University, Nanjing, China; ^2^School of Life Sciences, Anhui Normal University, Wuhu, China

**Keywords:** fungal-specific transcription factor, *Valsa pyri*, virulence, RNA-seq, differentially expressed genes

## Abstract

*Valsa pyri* is the causal agent of pear canker disease, which leads to enormous losses of pear production in eastern Asian, especially China. In this study, we identified a fungal-specific transcription factor 1 (termed as VpFSTF1) from *V. pyri*, which is highly conserved in fungi. To characterize its functions, we generated mutant and complementation strains in *V. pyri* and found that *ΔVpFSTF1* mutants lost the ability to form fruiting bodies along with the reduced virulence. The radial growth of *ΔVpFSTF1* mutant was sensitive to increasing concentrations of hydrogen peroxide (H_2_O_2_) and salicylic acid (SA). Moreover, RNA-sequencing (RNA-Seq) analysis of wild-type (WT) and *ΔVpFSTF1* mutant strains was performed, and the results revealed 1,993 upregulated, and 2006 downregulated differentially expressed genes (DEGs) in the mutant. The DEGs were corresponding to the genes that are involved in amino acid metabolism, starch, and sucrose metabolism, gluconeogenesis, citrate cycle, and carbon metabolism. Interestingly, pathogen host interaction (PHI) analysis showed that 69 downregulated genes were related to virulence, suggesting that they might function downstream of VpFSTF1. Nine DEGs were further validated by quantitative reverse transcription-polymerase chain reaction (qRT-PCR), and the results were consistent with RNA-seq analysis. Furthermore, promoter regions were predicted, and VpFSTF1 binding activity was assessed. We demonstrated that five promoters are directly or indirectly targeted by VpFSTF1, including catalase-related peroxidase (VPIG_01209) and P450 family genes. Taken together, these findings indicate that VpFSTF1 is crucial for the virulence of *V. pyri* via direct or indirect regulation of downstream genes expression and lay an important foundation for understanding the molecular mechanism of *V. pyri* infection.

## Introduction

*Valsa pyri* is an ascomycete organism (Sordariomycetes and Diaporthales) from Valsaceae family that causes pear and apple canker disease with significant fruit yield losses (Yin et al., 2015). The disease represents a significant threat to pear and apple production in East Asia including China and often leads to tree death or failure of the entire orchard (Abe et al., 2007; Wang et al., 2013; Li et al., 2015; Yin et al., 2015). The pathogen invades its host through wounded tissues and forms canker lesions (Wang et al., 2014). The primary management of the disease includes strict cultivation management, chemical treatment (Cao et al., 2009; Wang et al., 2013), and removal of infected tissues with subsequent fungicide application (He et al., 2018b). *V. pyri* can penetrate systemically in xylem and phloem tissues and infect the host at any time of the year, leading to many challenges in its control (Abe et al., 2007). *V. pyri* is a necrotrophic pathogen that may secrete many cell wall-degrading enzymes and peroxidases, which may help to facilitate the infection and colonization of host bark (He et al., 2018b). Overall, the *V. pyri* infection process is complex and involves numerous proteins, whose expression is usually orchestrated by transcription factors (TFs).

By regulating gene expression as activators or repressors, TFs have crucial roles in eukaryotic cells (Chung et al., 2013). Approximately 80 families of TFs have been found in fungi and function in various processes, including amino acid metabolism, vitamin synthesis, sugar metabolism, gluconeogenesis, respiration, meiosis, mitosis, chromatin remodeling, peroxisome proliferation, nitrogen utilization, pleiotropic drug resistance, and stress response (MacPherson et al., 2006; Shelest, 2017). Importantly, many TFs are crucial for fungal pathogenicity, for example, in *Magnaporthe grisea*, homeobox TFs are essential for conidiation and appressorium development ultimately affecting its pathogenicity (Kim et al., 2009; Zhang et al., 2009). AbVf19 TFs are required for virulence in *Alternaria brassicicola* (Srivastava et al., 2011). In *Valsa mali*, VmSeb1 TFs regulate growth, development and virulence (Wu et al., 2018). In *Verticillium dahlia*, VdMcm1 regulates conidiation, microsclerotium formation, pathogenicity, and secondary metabolism (Xiong et al., 2016).

Despite the large number of TFs in fungi, a great percentage of these factors are C6 Zn cluster (Zn_2_Cys_6_), C_2_H_2_-like Zn finger (C_2_H_2_) and homeodomain-like proteins (Luo et al., 2016; Shelest, 2017). All Zn_2_Cys_6_ TFs and a few C_2_H_2_ TFs are fungal-specific TFs that contain a fungal-trans domain, which is a typical feature of fungal-specific TFs (Shelest, 2017). Zn_2_Cys_6_ TFs contain a DNA-binding domain with six cysteine residues that together coordinate two zinc atoms and a fungal_trans domain, have been well studied in many fungi (MacPherson et al., 2006). However, only a few Zn_2_Cys_6_ TFs are crucial for virulence. For example, AbPf2 and its orthologs regulate effector gene expression to control fungal virulence (Cho et al., 2013; Rybak et al., 2017). In addition, fungal-specific TF Vdpf and VdFTF1 influence pathogenicity in *V. dahliae* (Luo et al., 2016; Zhang et al., 2017). EBR1 affects virulence and apical dominance of the hyphal tip in *Fusarium graminearum* (Zhao et al., 2011). Zcf15 and Zcf29 are required for the virulence of human pathogen *Candida albicans* (Issi et al., 2017). The Zn_2_Cys_6_ cluster domain binds as a dimer to CGG triplets that occur in everted, inverted, and direct repeats (MacPherson et al., 2006). Reports to date for this superfamily have mostly focused on crop pathogens or human pathogens, whereas there are few reports on woody plant pathogens.

*Valsa pyri* is mainly woody plant pathogen that infects the branches or trunks of pear and apple trees. Although various genes encoding TFs have been annotated in the *V. pyri* genome and transcriptome (Yin et al., 2015; He et al., 2018b), only VpCRZ1, which is a C_2_H_2_ Zn finger TF, has been characterized (He et al., 2016). Due to the importance of fungal-specific TFs in fungi, elucidation of their roles in the woody pathogen *V. pyri* will offer new insight into this TF family. Based on transcriptome analysis of two *V. pyri* isolates (He et al., 2018b), we observed that the expression levels of several Zn_2_Cys_6_ TF-encoding genes were induced in the infection stage. Since we considered that these TFs may be involved in the virulence of *V. pyri*, we selected one gene, *VpFSTF1* (Fungal Specific Transcription Factor, KUI53834.1), whose orthologs have not been characterized in other fungi. Using polyethylene glycol (PEG)-mediated transformation, we generated deletion mutant and complementation strains. The deletion mutant showed reduced virulence in pear and sensitivity to H_2_O_2_ and SA. Furthermore, RNA-seq analysis was performed, and several downstream genes were confirmed using qRT-PCR and Y1H. This study contributes in understanding of mechanism regulated by a novel Zn_2_Cys_6_ TF, VpFSTF1, a key regulator of virulence in *V. pyri*.

## Materials and Methods

### Bioinformatic Identification of VpFSTF1

The sequence of the gene encoding VpFSTF1 was obtained from the transcriptome (He et al., 2018b). The protein sequences of Zn_2_Cys_6_ TFs in *V. pyri* were archived using the HMMSCAN program from the HMM software suite HMM 3.0, and one protein-encoding gene (KUI53834.1) unregulated in the infection stage was chosen for further study. We obtained its orthologs in other fungi using BLASTP with the National Center for Biotechnology Information (NCBI) database^1^ and downloaded the corresponding sequences from *Theiervia terrestris* (XP_003653580.1), *Thermothelomyces thermophile* (XP_003660880.1), *Madurella mycetomatis* (KXX79220.1), *Neurospora crassa* (XP_011394332.1), *Podospora anserine* (CDP26737.1), *Podospora comate* (VBB76835.1), *Coniochaeta ligniaria* (OIW32290.1), *Sporothrix insectorum* (OAA67115.1), *Magnaporthe grisea* (ELQ404431.1), *Coniella lustricola* (PSS05266.1), *Diaporthe ampelina* (KKY30034.1), and *V. mali* (KUI68526.1) (McGinnis and Madden, 2004). Sequence alignment was analyzed with Clustal W (Thompson et al., 1994), and a phylogenetic tree was constructed using the neighbor-joining method in MEGA 7.0 software (Kumar et al., 2016). Confidence levels were obtained from a bootstrap test replicated 1000 times. The protein domains analyzed using SMART website^2^.

### Fungal Strain Culture and Fruiting Body Induction

*Valsa pyri* wild-type (WT) strain Vp297 was used. The WT and all the transformant strains generated in this study were regularly cultured on potato dextrose agar medium (PDA, 20% peeled potato, 2% dextrose, and 1.5% agar) at 25°C in the dark. Mycelia were cultured in potato dextrose broth (PDB, 20%, peeled potato and 2% sucrose) medium; after 48 h, the mycelia were collected and used for RNA and DNA extraction. Czapek Dox medium (30 g/L sucrose, 2 g/L NaNO_3_, 0.5 g/L MgSO_4_-7H_2_O, 0.5 g/L KCl, 0.02 g/L FeSO_4_⋅7H_2_O, and 1 g/L K_2_HPO_4_) was used for analysis of mutant growth on various carbon sources. A 5-mm mycelial agar plug was cut from the edges of 2-day-old colony cultures and used in stress and virulence inoculation assays.

The deletion mutants generated were characterized regarding to developmental and morphological features. WT, deletion mutant (K-29 and K-50), and complementation (C-140 and C-141) strains were cultured on PDA medium and incubated at 25°C under a fluorescent cycle of 16 h light/8 h dark for 15 days to promote fruiting body formation. Each experiment was repeated at least three times.

### Generation of the *ΔVpFSTF1* Deletion Mutant

The WT genomic DNA was extracted using the cetyltrimethylammonium bromide (CTAB) protocol (Umesha et al., 2016). Mutant alleles were constructed using double joint polymerase chain reactions (PCRs) (He et al., 2016). Upstream and downstream fragments were amplified using the 1/2 and 3/4 primer pairs (Supplementary Table S2), respectively. A cassette containing the hygromycin phosphotransferase gene (*hph*) was amplified using the 5/6 primer pair. Mutant gene constructs of the three PCR products were utilized as templates at a ratio of 1:3:1 and amplified using 1/4 primer pair. The deletion mutant recombinant construct was directly transformed into WT strain protoplasts using an improved PEG-mediated *V. pyri* transformation protocol (He et al., 2016). Stable transformants were initially screened on PDA medium supplemented with 50 mg/L hygromycin B. The *VpFSTF1* open reading frame (ORF) was then screened for genomic PCR using the 9/10 primer pair. Determination of a successful deletion in the *V. pyri* genome was performed using the 5/6 primer pair. Allele site replacement was confirmed using the 6/7 and 5/8 primer pairs. The transcript levels of the transformants were determined by qRT-PCR using the 17/18 primer pairs.

### Generation of the Complementation Strain

To obtain the complementation strain, we ligated *VpFSTF1* gene to vector pFL2 (Li et al., 2018) to generate a construct that harbors the target gene driven by the strong promoter *RP27* and also contains a neomycin resistance gene. To generate the *VpFSTF1* fusion pFL2:*VpFSTF1*:GFP, we amplified *VpFSTF1* ORF using the 11/12 primer pair. Briefly, the coding region for the C-terminus of *VpFSTF1* was fused to that of enhanced green fluorescent protein (EGFP) using double-joint PCR with the 13/14 and 15/16 primer pairs. The resulting PCR product was ligated into *Xho*I-digested *pFL2* and then transformed into *Escherichia coli* DH5ɑ cells. The plasmid was extracted using Plasmid Mini Kit 1 (Omega Bio-tek, Inc., 400 Pinnacle Way, Suite 450 Norcross, GA 30071). The plasmid was confirmed by PCR using the 15/16 primer pair (Supplementary Table S1) and by sequencing (GenScript, Nanjing, China) and then transferred to *V. pyri*. One hundred fifty transformants were obtained by screening with 75 mg/L G418 on PDA, and several positive transformants were confirmed by genomic PCR using the 7/8 primer pair and qRT-PCR using the 17/18 primer pair (Supplementary Table S1).

### RNA Extraction and Quantitative RT-PCR

Total RNA was extracted from WT, Δ*VpFSTF1*, and *VpFSTF1*com using the RNAsimple Total RNA kit (Tiangen, Beijing, China). Total RNA concentrations were measured using a spectrophotometer (Nanodrop ND-1000), and quality was determined by agarose gel electrophoresis. The RNA was inoculated at 42°C for 2 min to digest DNA, and cDNA was synthesized with PrimeScript reagent kit (TaKaRa). qRT-PCR was conducted using an ABI Prism 7300 Fast Real-Time PCR System (Applied Biosystems) with SYBR Premix ExTaq (TaKaRa). Reaction Ct values were determined, and expression levels were calculated using 2^–ΔΔ*Ct*^ (Livak and Schmittgen, 2001). For comparison with WT levels, the transcript levels of candidate genes were normalized to that of the *V. pyri actin* (KUI53217.1) gene. Eachexperiment was repeated at least three times.

### Gene Expression Pattern During Infection

To determine the transcriptional profile of *VpFSTF1* in the WT strain during pear infection, pear bark slices inoculated with WT mycelia were harvested at 0, 3, 6, 12, 24, and 48 hpi (hours post inoculation) and then frozen in liquid nitrogen and stored at −70°C. The pear bark slices that were initially inoculated with WT mycelia were set as 0 hpi. Total RNA from inoculated pear tissues was extracted using the CTAB-LiCl protocol (Gambino et al., 2010). Total RNA concentration and quality determinations, cDNA synthesis, and qRT-PCR were performed as described above. All experiments were repeated at least three times.

### Mutant Utilization of Carbon Sources

The growth of the deletion mutant was determined using three carbon sources: pectin, cellulose, and sucrose. Each carbon source was separately added (20 g/L) to Czapek Dox medium (lacking carbon source), and Czapek Dox medium lacking all carbon sources was employed as a control. The petri plates containing medium of three different carbon sources were inoculated with mycelial agar plugs and incubated at 25°C. Colony diameter was measured and recorded, and typical images were taken at 24 hpi. The experiment was repeated at least three times.

### Measurement of Stress Responses

To assess the sensitivity of the mutant strain to oxidative, hydrogen peroxide (H_2_O_2_) and salicylic acid (SA) stresses, mycelial agar plugs of the WT, Δ*VpFSTF1*, and *VpFSTF1*com strains were placed onto PDA medium supplemented with 0, 0.5, 1.0, 1.5 or 2.0 mM H_2_O_2_ or 0, 1, 2, 3, or 4 mM SA (Sigma-Aldrich). The plates were incubated at 25°C in the dark. Colony diameter was measured and photographed at 48 h for H_2_O_2_ and 24 h for SA. The experiment was repeated at least three times.

### Pathogenicity Assay

Fresh pear leaves and 1-year-old branches were used for this assay. The leaves and branches were initially wounded with needles and then inoculated with agar plugs cut from 2-day-old PDA cultures of the WT, Δ*VpFSTF1*, and *VpFSTF1*com strains. The inoculated leaves and branches were placed on clean trays and incubated at 25°C in the dark. Disease development on inoculated leaves and branches was observed daily during the incubation period. Lesion canker sizes were measured at 3 and 5 days post inoculation (dpi). The assay was repeated with at least eight leaves and ten branches in each treatment, and the data were analyzed using ANOVA.

### RNA Extraction, Library Construction, and RNA Sequencing

The WT strain Vp297 and the mutant K-50 were cultured on PDA medium for 2 days at 25°C in the dark, and then fresh fungal plugs were transferred in PDB for 36 h at 25°C. Total RNA extraction, concentration and quality determinations were carried out as described above. Three biological replicates were prepared for the WT and mutant. cDNA library preparation and sequencing were performed using the Illumina sequencing HiSeq^TM^ 2000/2500 platform (Illumina^TM^, San Diego, CA, United States) (He et al., 2018a). Clean reads were obtained by the removal of reads containing the adapter and low-quality reads from raw data (raw reads), and GC content was calculated from clean reads using the genome of *V. pyri* as a reference (Yin et al., 2015). The expression levels of each predicted gene were analyzed using HTSeq through the union model, and they were determined as reads per kilobase of exon model per million (RPKM) (Anders and Huber, 2010). Differentially expressed genes (DEGs) were identified from three biological replicates for both the mutant and Vp297 using DESeq R with a 1% false discovery rate (FDR) and *P* ≤ 0.05 cutoff (Anders and Huber, 2010). We performed gene ontology (GO) analyses using the GOseq package (Young et al., 2010; The Gene Ontology Consortium, 2017) as well as analyses of kyoto encyclopedia of genes and genomes (KEGG) pathways with the KEGG orthology (KO)-Based Annotation System (KOBAS) (Mao et al., 2005). The significant enriched GO terms were selected based on biological process and molecular function (MF) using *p*-value < 0.05 as the criterion. DEGs involved in virulence were analyzed using BLASTp with the pathogen-host interaction (PHI) database^3^, and virulence-related genes were obtained (Supplementary Table S3). To confirm whether *VpFSTF1* gene was knocked out, the reads from the wild type and K-50 were mapped to *V. pyri* genome via Hisat2 and visualized by IGV2.7.2 (Thorvaldsdottir et al., 2013).

### qRT-PCR Validation of Differentially Expressed Genes

qRT-PCR was performed to determine the molecular mechanisms associated with VpFSTF1-mediated regulation of virulence-related genes in *V. pyri*. WT and mutant total RNA in three biological replicates was extracted, and cDNA was synthesized as described above. The expression levels of DEGs in the WT and mutant strains were quantified using qRT-PCR (an ABI Prism 7300 Fast Real-Time PCR System (Applied Biosystems) with SYBR Premix ExTaq (TaKaRa). Ct values were exported, and levels of gene expression were calculated using 2^–ΔΔ*Ct*^ (Livak and Schmittgen, 2001). For comparison with WT levels, transcript levels of candidate genes were normalized to those of the *V. pyri actin* gene. The experiment was repeated at least three times.

### Yeast One-Hybrid Assay

Genes for yeast one-hybrid (Y1H) experiments were selected according to the results of PHI analysis. The promoters of these genes were considered a 1500-bp fragment upstream of ATG start codon. The promoter fragments were amplified using WT genomic DNA as a template and cloned into the pHIS2 (BD vector) plasmid, which had been linearized by *Sma*I digestion. TFs were cloned into a pGADT7 (AD vector) plasmid linearized by *Nde*I digestion. The plasmids were sequenced by GenScript Company, Nanjing, China. The pAD/VpFSTF1 construct containing the VpFSTF1 sequence and the pHIS2 reporter construct were co-transformed into AH109 Gold yeast cells. The cells were cultured on SD–Trp–Leu medium and then sub-cultured on SD–Trp–Leu–His medium amended with 10 mM 3-aminotriazole (Aldrich) with 10-fold gradient dilutions at 28°C for 3 days. The results were recorded, and the representative images were taken. Growth on SD–Trp–Leu–His medium containing 10 mM 3-aminotriazole indicated that the TFs were able to bind to the promoter region and stimulate gene expression (Mao et al., 2016; Miao et al., 2018).

## Results

### Identification and Transcript Expression Analysis of *VpFSTF1* During *Valsa pyri* Infection of Pear

**FIGURE 1:**
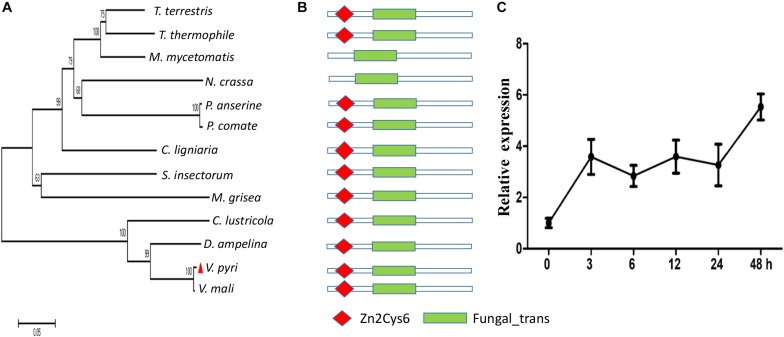
Identification and transcript expression analysis of *VpFSTF1* during *V. pyri* infection of pear. **(A)** Phylogenetic analysis of fungal VpFSTF1 proteins. Fungal-specific transcription factor sequences were downloaded from the NCBI database. The sequences were aligned using the Clustal W tool, and a phylogenetic tree was constructed by the neighbor-joining method using MEGA 7.0 software. The confidence levels above the nodes were obtained from 1000 bootstrap analysis. **(B)** Main domains of VpFSTF1. The domain architecture of VpFSFT1 and its orthologs were predicted with SMART. **(C)** Transcriptional expression analysis of *VpFSTF1* during *V. pyri* infection of pear. The relative levels of *VpFSTF1* in *V. pyri* were quantified by qRT-PCR and normalized to the transcript levels of the *V. pyri actin* gene. Fold changes were calculated by the 2^–ΔΔ*Ct*^ approach.

Previously, we found that seven Zn_2_Cys_6_ TF-encoding genes were upregulated during the infection phase (Supplementary Table S1), among which, a gene (KUI53834.1) was 4.8-fold higher in infected pear fruit at 24 dpi than the mycelial stage. This gene was selected for further study and designated as *VpFSTF1* (*V. pyri* Fungal Specific Transcription Factor 1). Phylogenetic analysis of the fungal orthologs showed that VpFSTF1 shared highly sequence similarity with its ortholog in *V. mali* and other species (Figure 1A). Furthermore, the protein domains analyzed using SMART revealed that VpFSTF1 contains a Zn cluster Zn2Cys6 domain and a fungal-trans domain, similar to its orthologs (Figure 1B). To determine its expression pattern during infection, we performed qRT-PCR analysis on infected samples at different time points (0, 3, 6, 12, 24, and 48 hpi). Expression of *VpFSTF1* was upregulated by threefold at 3 hpi compared to the time of initial invasion and at least fourfold at 48 hpi (Figure 1C). These results suggest that VpFSTF1 is a fungal-specific TF and that its expression was markedly induced in *V. pyri* during the initial infection stage of pear.

### Generation of the *ΔVpFSTF1* Mutant

In order to functionally characterize *VpFSTF1* in *V. pyri*, we generated a gene knockout mutant using an improved PEG-mediated fungal transformation protocol (He et al., 2016). The ORF of *VpFSTF1* was replaced by a hygromycin resistance cassette (*hph*) (Supplementary Figures S1Ai–iii). The *VpFSTF1* deletion mutants were initially screened on PDA medium containing 50 mg/L hygromycin B, and more than 200 primary transformants were obtained. To identify true knockout transformants, a partial DNA fragment of the *VpFSTF1* ORF was amplified by genomic PCR, with no amplification in one hundred thirty mutants (Supplementary Figure S1Bi). However, the *hph* fragment was successfully amplified in these mutants (Supplementary Figure S1Bii), indicating that the original gene was successfully deleted. To further confirm whether this process resulted in allele replacement, we performed genomic PCR using outer primers consisting of *hph* cassette primers and found that two independent strains (K-29 and K-50), but not the WT, showed positive bands, demonstrating successful deletion of the target gene in them (Supplementary Figures S1Biii,iv). Furthermore, we selected one mutant (K-50) to generate complementation strains and the results of genomic PCR showed that the gene was successfully restored in two independent transformants (C-140 and C-141) (Supplementary Figures S1Bi–iv). Accordingly, *VpFSTF1* transcripts were undetectable in the knockout mutants, whereas the gene was overexpressed in the complementation strains (Supplementary Figure S1C). Taken together, these results demonstrate that the gene was successfully knocked out.

### VpFSTF1 Is Required for the Formation of Fruiting Bodies

To determine whether VpFSTF1 plays a role in growth of *V. pyri*, we cultured WT, Δ*VpFSTF1* (K-29 and K-50) and *VpFSTF1*com (C-140 and C-141) on media containing pectin, sucrose, and cellulose. None differences were found among the WT, the deletion mutants and the complementation strains on these carbon sources (Supplementary Figures S2A,B). These results suggest that VpFSTF1 is not involved in growth of *V. pyri* on media amended with different carbon sources. However, when mutants were cultured on PDA medium, fruiting bodies did not form, unlike the complementation and WT strains (Supplementary Figure S2C). These results indicate that VpFSTF1 plays a crucial role in the formation of fruiting bodies in *V. pyri*.

### The *Valsa pyri ΔVpFSTF1* Mutant Is Hypersensitive to Oxidative Stress

**FIGURE 2:**
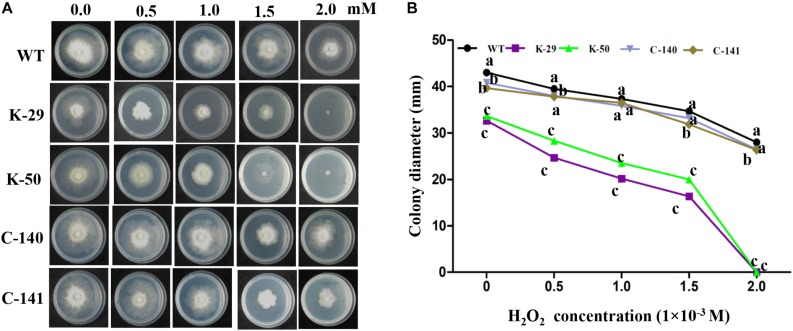
Hypersensitivity of the *ΔVpFSTF1* mutant to H_2_O_2_. **(A)** Effect of oxidative stress on colony growth. Wild-type (WT), *ΔVpFSTF1* (K-29 and K-50) deletion mutant and VpFSTF1com (C-140 and C-141) complementation strains were grown on PDA medium supplemented with indicated H_2_O_2_ concentrations and incubated at 25°C for 48 h. Representative photographs are shown. **(B)** Radial growth of different strains on PDA medium under oxidative stress compared with growth on PDA without oxidative stress. Colony diameters were measured at 48 h. Different letters indicate significant differences (*P* < 0.05, ANOVA).

To determine whether VpFSTF1 is involved in the response to oxidative stress, we cultured the mutant, WT and complementation strains on PDA medium amended with 0, 0.5, 1, 1.5, and 2 mM H_2_O_2_ and measured colony diameters. Compared with WT strain, colony growth of deletion mutants showed negative relation with increased concentration of H_2_O_2_, and colony growth was completely inhibited at 2.0 mM H_2_O_2_ compared with WT. The colony growth of the complementation strain was comparable to WT at indicated H_2_O_2_ concentrations (Figure 2). These results show that VpFSTF1 is involved in the response to oxidative stress in *V. pyri*.

### VpFSTF1 Is Essential for Salicylic Acid Elimination by *Valsa pyri*

**FIGURE 3:**
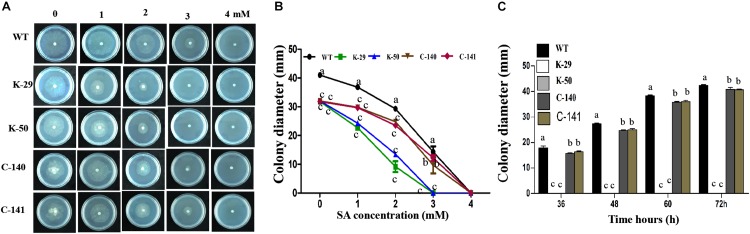
Effects of *VpFSTF1* gene deletion on the *V. pyri* SA stress response. **(A)** WT, *ΔVpFSTF1*, and *VpFSTF1*com strains were grown on PDA medium amended with 0, 1, 2, 3, and 4 mM SA and incubated at 25°C in the dark for 24 h. Representative photographs are shown. **(B)** Radial growth of the strains on PDA supplemented with different concentrations of SA compared with PDA without SA. Colony diameters were measured at 24 h. **(C)** Inhibition of colony growth on PDA with 4 mM SA at indicated time points. Colony diameter was measured at indicated time points. Bars indicate the standard deviation of the mean of three replicates. Different letters indicate significant differences (*P* < 0.05, ANOVA). The experiment was repeated three times with similar results.

To assess whether VpFSTF1 is involved in the response to SA stress, we measured colony diameters of the mutant, WT and complementation strains grown on PDA medium amended with 0, 1, 2, 3, and 4 mM SA (Figure 3A). The results for radial growth demonstrate that the mutant was more sensitive to SA than the WT, with colony growth inhibited at 3 and 4 mM SA after 24 h (Figure 3B). Colony growth of the complementation strain was comparable to the WT at all tested concentrations. To further evaluate the role of VpFSTF1 in response to SA stress, culture plates with 4 mM SA were incubated up to 72 h, and colony growth of the mutant was completely inhibited, though the diameters of the WT and complementation strain colonies gradually increased (Figure 3C). These results suggest that VpFSTF1 is involved in SA elimination *in vitro* and possibly also in pear trees.

### VpFSTF1 Is Required for Virulence

**FIGURE 4:**
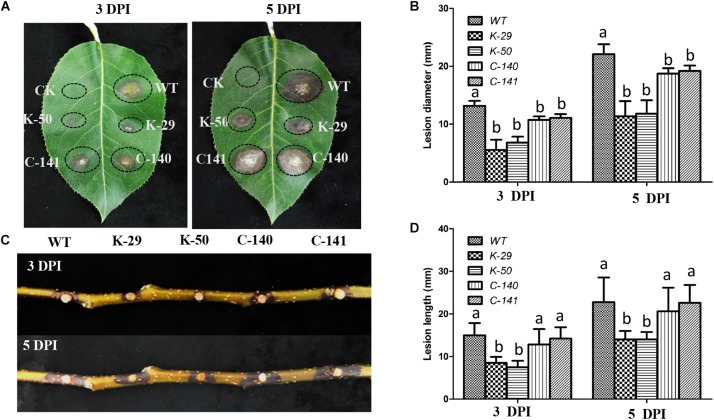
Phenotypes of pear leaves and branches inoculated with the *ΔVpFSTF1* deletion mutant of *V. pyri*. **(A)** Abaxial surfaces of pear leaves were inoculated with mycelial agar plugs of WT, *ΔVpFSTF1*, and *VpFSTF1*com strains. The inoculated leaves were incubated at 25°C in the dark. Lesions were photographed at 3 and 5 dpi, respectively. **(B)** Lesion diameters at inoculated sites on the leaves. **(C)** Wounded 1-year-old branches were inoculated with mycelial agar plugs of WT, *ΔVpFSTF1*, and *VpFSTF1*com strains and incubated at 25°C in the dark. The lesions were photographed at 3 and 5 dpi, respectively. **(D)** Inoculated lesion lengths on pear branches. Bars indicate the standard deviations of means of three replicates. Different letters indicate significant differences (*P* < 0.05, ANOVA).

To determine whether VpFSTF1 plays a role in disease development, virulence assays were performed on pear leaves and branches using the WT, Δ*VpFSTF1*, and *VpFSTF1*com strains, and lesion sizes were quantified. Compared with the WT lesion diameters, the mutant lesion diameters were slightly reduced on pear leaves at 3 dpi and significantly reduced at 5 dpi (Figures 4A,B), and lesions on 1-year-old pear branches caused by inoculation with the mutant were significantly shorter at both 3 and 5 dpi (Figures 4C,D). The virulence of the complementation strains was similar to the WT (Figure 4). These results indicate that VpFSTF1 plays a significant role in regulating virulence in *V. pyri*.

### Differentially Expressed Genes and Functional Annotations

**FIGURE 5:**
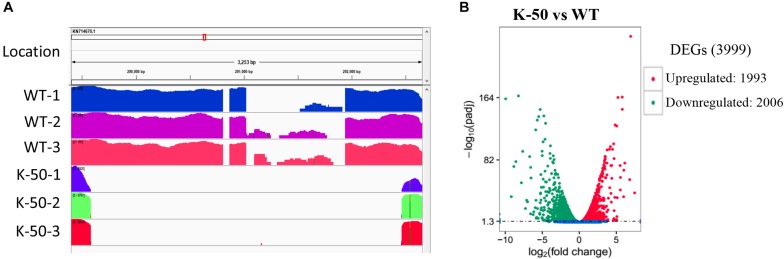
Gene characterization and analysis of differentially expressed genes (DEGs). **(A)** The reads from the wild type (Vp297) and K-50 mutant mapped to the fragment including the knockout site. **(B)** Volcano plot of DEGs between K-50 and WT. The *x*-axis represents the log_2_-fold change. The *y*-axis represents –log10 adjustments (padj). The red spots indicate upregulated DEGs, and the green spots indicate downregulated DEGs.

To investigate transcriptional changes due to gene knockout, we selected the WT and the mutant K-50 for RNA-seq analysis. We prepared mycelia of the WT (Vp297) and mutant in three biological replicates. The RNA-seq analysis produced 248,348,202 paired-end reads, and the raw data were deposited in SRA database (Accession numbers PRJNA588311). After the removal of adapters and low-quality reads, 232,175,488 bp of clean data were acquired. The Q30 value was more than 93.45%. The genome of *V. pyri* was used as a reference (Yin et al., 2015). Clean reads were mapped to this genome with a ratio ranging from 92.48 to 96.34%. Approximately 92.2 to 95.96% of the total mapped reads were uniquely aligned. Multi-aligned reads were removed, and only unique reads were used for further analysis. These results indicate the good quality of our *V. pyri* RNA sequencing. To examine whether *VpFSTF1* gene was knocked out, the reads from the wild type and K-50 samples were mapped to deletion region. We observed that there are no reads from K-50 samples mapped on the deletion part, while abundant reads from the WT strian were detected in this region (Figure 5A), further confirming that *VpFSTF1* gene was successfully deleted from the genome. Furthermore, we analyzed DEGs between the K-50 mutant and WT using 1% FDR and *P* ≤ 0.01 for upregulation and downregulation as the criteria for defining DEGs. The DEGs were further validated by RT-qPCR (Supplementary Figure S3 and Supplementary Table S4), the results were highly consistent with RNA-seq analysis with a high Pearson coefficient (*R* = 0.899) (Supplementary Table S4). Finally, we identified 3999 significant DEGs between K-50 and Vp297, including 1993 upregulated and 2006 downregulated DEGs in the K-50 mutant (Figure 5B).

### Gene Ontology and Kyoto Encyclopedia of Genes and Genomes Pathways of Differentially Expressed Genes

**FIGURE 6:**
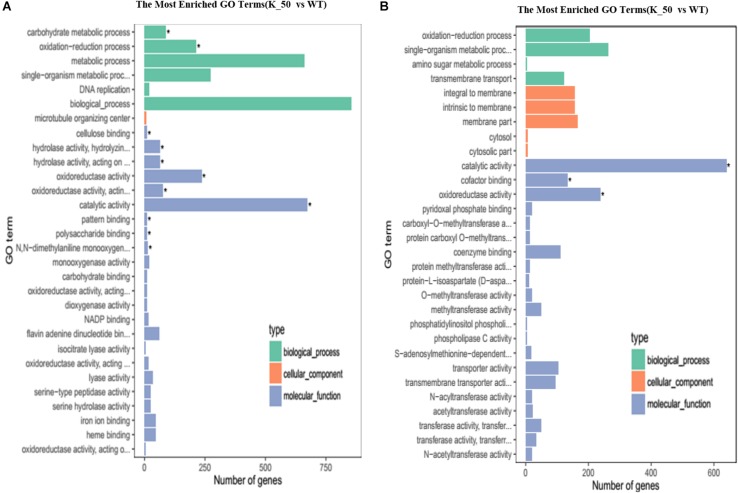
Gene ontology (GO) analysis of DEGs. **(A)** The most enriched GO terms. The downregulated DEGs of each category are shown on the graph. **(B)** The upregulated DEGs of each category are presented on the graph as related to BP, CC, and MF. BP, biological process; CC, cellular component; MF, molecular function. ^∗^ indicates the significantly enriched terms.

Gene ontology annotation was performed to reveal the function of DEGs. For downregulated DEGs, biological processes, microtubule organizing center, and catalytic activity were abundant in biological process (BP), cellular component (CC), and MF, respectively (Figure 6A). For upregulated DEGs, single organism metabolic processes, membrane part, and catalytic activity were the most plentiful categories in BP, CC, and MF, respectively (Figure 6B). To identify which MF or biological process is affected in the deletion mutant, we performed GO enrichment analysis. The results showed that cellulose binding, catalytic activity, oxidoreductase activity, polysaccharide binding, and pattern binding related genes were significantly enriched (Figure 6A).

**FIGURE 7:**
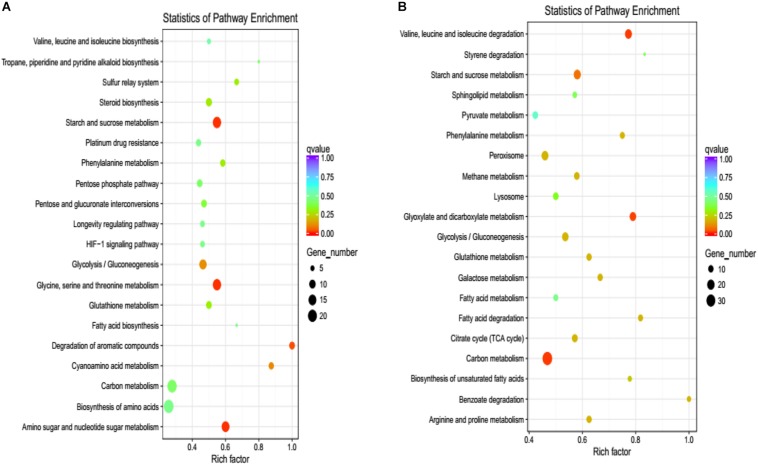
KEGG pathway enrichment of DEGs. **(A)** The graph shows enriched KEGG terms for downregulated DEGs influenced by the gene deletion. **(B)** The pathways of enriched KEGG terms for upregulated DEGs influenced by the gene deletion. Rich factor is the ratio of the differentially expressed gene number to the total gene number in a certain pathway.

To confirm which pathway was interrupted in the deletion mutant, we performed KEGG analysis to categorize the most enriched pathways. Downregulated DEGs involving amino acid metabolism, starch, and sucrose metabolism, gluconeogenesis, the citrate cycle, and carbon metabolism were enriched, and their functions are associated with energy production, growth, and development in *V. pyri* (Figure 7A). For upregulated DEGs, pathways related to the sulfur relay system, steroid biosynthesis, and the pentose phosphate pathway were enriched (Figure 7B).

### qRT-PCR Validation of Differentially Expressed Genes

**FIGURE 8:**
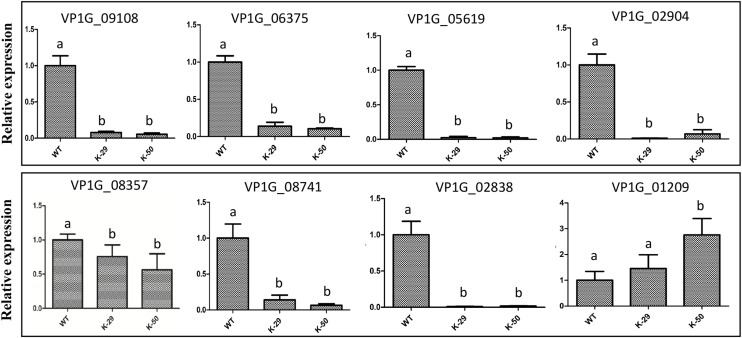
qRT-PCR analysis of the representative DEGs. Expression levels of isotrichodermin C-15 hydroxylase (VP1G_08741), MFS transporter (VP1G_06375), trichodiene oxygenase (VP1G_05619), NPP1 (VP1G_02838), pisatin demethylase (VP1G_08357), fumitremorgin c monooxygenase (VP1G_09108), catalase-related peroxidase (VP1G_01209), and TOX (VP1G_02904). The expression level of each gene was analyzed by qRT-PCR and normalized to actin expression, and the relative expression ratio was calculated as 2^–ΔΔ*Ct*^ compared to that of WT. Different letters indicate significant differences, *P* < 0.05, ANOVA).

Because VpFSTF1 has important roles in virulence and H_2_O_2_ stress, we deduced that virulence-related genes or H_2_O_2_ scavenging-related genes may be controlled by TFs. To evaluate whether the DEGs obtained are involved in virulence or H_2_O_2_ stress, we annotated the DEGs with PHI database via blast searches. According to our analysis, sixty-nine DEGs may have important roles in virulence or H_2_O_2_ stress (Supplementary Table S3). For example, nine DEGs may involve in virulence, including isotrichodermin C-15 hydroxylase (VP1G_08741, KUI61556.1), MFS transporter (VP1G_06375, KUI59140.1), trichodiene oxygenase (VP1G_05619, KUI58327.1), NPP1 (VP1G_02838, KUI55454.1), pisatin demethylase (VP1G_08357, KUI61188.1), fumitremorgin c monooxygenase (VP1G_09108, KUI61983.1), catalase-related peroxidase (VP1G_01209, KUI53753.1), TOX (VP1G_02904, KUI55504.1), and oxalate–CoA ligase (VP1G_02119, KUI54754.1). To confirm whether these 9 genes are regulated by VpFSTF1, we performed a RT-qPCR validation. We found that except VP1G_02119 (has a very low expression level, data not shown) and VP1G_01209, all the rest 7 genes were significantly downregulated in the deletion mutant, indicating that the transcriptomic data were reliable (Figure 8). These results suggest that VpFSTF1 can positively control the level of virulence-related gene expression to affect virulence in *V. pyri.*

### Transcription Factor Promoter-Binding Activity

**FIGURE 9:**
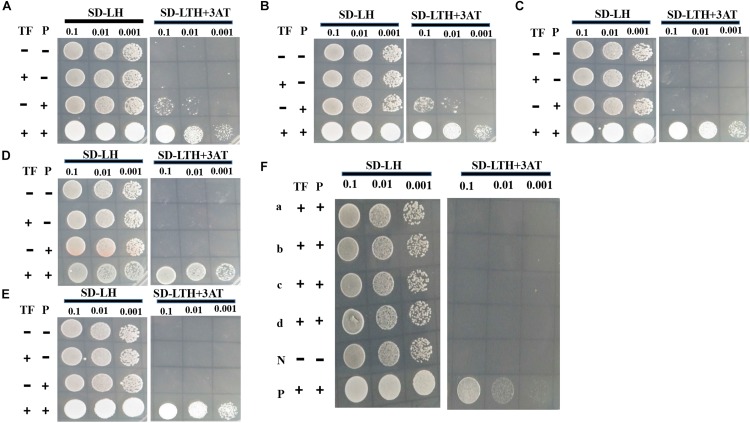
VpFSTF1 (TF) binding activity test using yeast one-hybrid (Y1H) assays. Genes were selected from PHI analysis. **(A)** VP1G_08357, Pisatin demethylase, **(B)** VP1G_05619, trichodiene oxygenase, **(C)** VP1G_02119, oxalate-CoA ligase, **(D)** VP1G_01209, catalase-related peroxidase, and **(E)** VP1G_09108, fumitremorgin C monooxygenase. **(F)** (a) VP1G_02904, TOX, (b) VP1G_08741, isotrichodermin C-15 hydroxylase, (c) VP1G_06375, MFS transporter, and (d) VP1G_02838, NPP1. Yeast transformants were grown on SD/-Leu/His (SD-2) and selected on SD/-Trp/-Leu/-His (SD-3 + 3AT). The plasmid pGADT7 *VpFSTF1* (AD-*VpFSTF1*, TF) and pHIS2 (P) plasmids containing each promoter of the above genes were cotransformed into the yeast AH109 Gold. The positive control strain contains 53 m: pGADT7, and p53:pHis, AD-empty (–) and BD-empty (–) plasmids were used as negative controls. Yeast transformants were grown on SD/-Leu/His (SD-2) and selected on SD/-Trp/-Leu/-His (SD-3 + 3AT). The plasmid pGADT7 *VpFSTF1* (AD-*VpFSTF1*, TF) and pHIS2 (P) plasmids containing each promoter of the above genes were co-transformed into the yeast AH109 Gold. AD-empty (–) and BD-empty (–) plasmids were used as negative controls. The cultures were diluted to 1–10^–3^ by 10-fold gradient dilution (OD_600_ = 0.5). Cultures were diluted to 1–10^–3^ by 10-fold gradient dilution (OD_600_ = 0.5). The results were obtained after 3 days of growth at 28°C, and typical images were taken.

To determine whether gene transcript levels are directly controlled by VpFSTF1, TF promoter-binding activity was assessed by Y1H using putative promoter (approximately 1500 bp upstream of start codon) of above nine DEGs (Supplementary Table S3). The TF-protein interaction results showed that VpFSTF1 was able to bind to the promoter regions of the genes encoding pisatin demethylase, trichodiene oxygenase, oxalate-CoA ligase, catalase-related peroxidase, and fumitremorgin C monooxygenase *in vivo*, suggesting that these genes may be downstream of VpFSTF1 in the regulation of virulence or H_2_O_2_ stress (Figures 9A–E). In contrast, the remaining promoters may not be directly controlled by VpFSTF1 (Figure 9F). These results indicate that VpFSTF1 is necessary for controlling virulence-related genes expression in *V. pyri.*

## Discussion

*Valsa pyri* is a woody pathogen that causes pear or apple canker disease (Yin et al., 2015), which represents a significant threat to pear and apple production (Abe et al., 2007; Wang et al., 2013; Yin et al., 2015). To infect a host pear or apple tree, the pathogen must overcome the stresses or resistance produced by the host plant, such as ROS (reactive oxygen species) and SA stresses, PTI (pathogen-triggered immunity) and ETI (effector-triggered immunity) (Jones and Dangl, 2006; Shamrai, 2014). In the invasion period, transcription of the pathogens will be alerted to increase the production of proteins that aid in invasion (Cho et al., 2013; Zhang et al., 2017; He et al., 2018b). TFs regulate gene expression during this process, which is required for infectious growth or infection (Zhao et al., 2011; Cho et al., 2013; Rybak et al., 2017; Zhang et al., 2017). Many Zn_2_Cys_6_ TFs are involved in virulence in fungi. However, no orthologs of *VpFSTF1* have studied. Moreover, *V. pyri* hosts are woody plants, and this pathogen secretes many cell wall-degrading enzymes, peroxidases, and amino acid transporters, which mainly participate in degrading host nutrients or ROS (He et al., 2018b). Nonetheless, as limited experimental studies have performed to date, our findings will enrich the knowledge regarding invasion mechanism of this pathogen.

Because orthologs of VpFSTF1 are not well studied in other fungi, a *VpFSTF1* deletion mutant was generated to explore its function. The phenotypic characterization showed that the mutant exhibited an inability to form fruiting bodies but no significant reduction in growth (Supplementary Figure S1). Importantly, the virulence was significantly reduced (Figure 4). Furthermore, *VpFSTF1* deletion caused greater sensitivity to host factors elicited in response to infection, such as H_2_O_2_ and SA. These results provide proof that VpFSTF1 is important for virulence. Moreover, these results indicate that VpFSTF1 is a multifunctional fungal-specific TF in *V. pyri*. The virulence reduction phenotype of the *VpFSTF1* mutant is similar to the many reported Zn2Cys6 TFs mutants, including VdPf (Luo et al., 2016), AbPf2 (Cho et al., 2013), VdFTF1 (Zhang et al., 2017), TPC1 (Galhano et al., 2017), MoCOD1, and MoCOD2 (Chung et al., 2013). Similar but not the same, TPC1 can control the growth of mycelia (Galhano et al., 2017) while VdFTF1 has no impact on vegetative growth, mycelial pigmentation and conidial morphology (Zhang et al., 2017) and VdPf can influence conidial production and melanized microsclerotium formation (Luo et al., 2016). The phenotype of the *VpFSTF1* mutant was mostly similar to that of the *VdPf* mutant in *V. dahliae*. However, these TFs are not orthologs of VpFSTF1. Unexpectedly, the phenotypes of *VpFSTF1* mutants were similar to that of a C_2_H_2_ TF VpCRZ1 mutant, suggesting that both TFs possibly regulate genes controlling fruiting body formation and pigment formation in *V. pyri* (He et al., 2016). Taken together, the results of our study contribute to further understanding of the Zn_2_Cys_6_ TFs family.

Deletion of *VpFSTF1* from *V. pyri* resulted in less virulence than WT (Figures 4A–D), which is consistent with previous studies on the filamentous fungi *M. grisea* (Choi et al., 2009), *A. brassicicola* (Cho et al., 2013), and *V. dahliae* (Luo et al., 2016), though the mechanism of the reduced virulence was still unclear. In our study, the oxidative stressors H_2_O_2_ and SA inhibited colony growth to various degrees. The sensitivity of deletion mutants to oxidative stress increased with increasing H_2_O_2_ concentration (Figures 2A,B), which is consistent with previous studies on *M. grisea, F. graminearum* (Nikolaou et al., 2009), and *Ustilago maydis* (Molina and Kahmann, 2007). Additionally, the sensitivity of the mutant to SA stress also increased with concentration (Figures 3A–C), consistent with previous studies on *Phytophthora capsici* (Candela et al., 2010) and *Eutypa lata* (Amborabé et al., 2002). In response to pathogenic infection, plants can vary the internal environment of the apoplast, xylem and phloem, including production of ROS and phytoalexin, and alteration of pH (Bhattacharjee, 2005). Some TFs, such as the bZIP TFs (MoAP1 and YAP1), are essential for ROS and other defense responses in host plants (Cessna et al., 2000; Molina and Kahmann, 2007; Guo et al., 2011). The reduced virulence of the deletion mutant might result from reduction of ROS suppression activity or expressions of virulent genes.

Because the function of VpFSTF1 has not been reported in other fungi, how it regulates virulence or other phenotypes remains unclear. To evaluate the level of transcription promoted by TFs, we used RNA-Seq to illustrate global gene transcript changes in the mutant. DEGs were obtained by bioinformatics analysis and further validated by RT-qPCR (Supplementary Figure S3 and Supplementary Table S4). The results were highly consistent with RNA-seq analysis with a high Pearson correlation (*R* = 0.899) (Supplementary Table S4). Among these DEGs, the most enriched GO terms between the mutant and WT were similar to the results of the transcriptomic analysis for the DEGs, highlighting catalytic activity, hydrolase activity and oxidoreductase activity. These GO terms were also abundant in *Aspergillus cristatus* and *A. brassicicola* (Cho et al., 2013; Tan et al., 2018). Combined with the phenotypes of the deletion mutants, these enrichments indicated that VpFSTF1 also plays important roles in H_2_O_2_ manipulation which may involve in response plant immune. However, there were also many oxidoreductase encoding genes upregulated in the mutant. We deduced that these genes may supplement the loss-function caused by the deletion of *VpFSTF1.* Regardless, for GO and KEGG enrichment, there was insufficient information indicating involvement in virulence.

Thus, to obtain genes involved in virulence, the DEGs were annotated using the PHI database. Finally, 69 proteins were obtained, including protein kinases, secreted proteins, catalase-1, and cell wall-degrading enzymes (Supplementary Table S3). Multiple genes are involved in virulence, such as MFS transporter (Choquer et al., 2007), Dicer-like protein 1 (Feng et al., 2017) and Catalase (Hernandez et al., 2010). And their orthologs VP1G_06375 (KUI59140.1), VP1G_04205 (KUI56844.1), VP1G_02970 (KUI55594.1) are also modulated by VpFSTF1, indicating that VpFSTF1 may be a key regulator. In our study, we found multiple genes are downregulated in the deletion mutant. Based on our analysis, several TFs such as zas1 (VP1G_00919, KUI53557.1), klf1 (VP1G_06378, KUI59144.1), and ZIC 5 (VP1G_06489, KUI59194.1) (Supplementary Table S3) are responsive to VpFSTF1 gene deletion, further implying that VpFSTF1 may function as a hub of gene regulatory network. However, there are many proteins with no clear functions in various fungi (Supplementary Table S3). For example, MgNLP was not required in *Mycosphaerella graminicola* (Motteram et al., 2009), but CoNLP could impair infection in *Colletotrichum orbiculare* (Azmi et al., 2018). The hypothetical proteins may be involved in virulence of *V. pyri*, but further analysis need detail characterization in the future. These results of our analysis demonstrated that VpFSTF1 might control many virulence-related genes and development-related genes, leading to virulence reduction in the *VpFSTF1* deletion mutant. These analyses also provide clues for further studies on the pathogenic mechanism of *V. pyri*.

We also examined the molecular mechanism by which VpFSTF1 regulates target genes using qRT-PCR and Y1H. The results demonstrate that nine genes are directly or indirectly regulated by VpFSTF1. Among them, pisatin demethylase, fumitremorgin C monooxygenase and trichodiene oxygenase belong to the P450 family and are important for pathogenic fungus infection (Funnell and VanEtten, 2002; Siewers et al., 2005; Alexander et al., 2008; Zhang et al., 2012). Orthologs of NPP1 in different fungi have various roles (Motteram et al., 2009; Azmi et al., 2018). Although the level of NPP1 expression was significantly reduced in the deletion mutant, further characterization is needed to confirm its roles in *V. pyri*. Moreover, the promoter-binding experiment was performed *in vivo*, and VpFSTF1 can bind several gene promoters directly. P450 family play important roles in pigment synthesis in *VdPf* deletion mutant (Luo et al., 2016) and *VpFSTF1* mutants, indicating that Zn2Cys6 TFs may have conserved roles in pigment deposition. VpFSTF1 could directly control the catalase-related peroxidase responding to H_2_O_2_ stress. However, the studies for these genes involving in virulence in *V. pyri* should be carried out in the future. Therefore, these results indicate that VpFSTF1 controls expression of virulence-related genes by directly or indirectly binding to their promoters, and further affects fungal virulence.

## Conclusion

In conclusion, VpFSTF1, a novel fungal-specific TF with a Zn_2_Cys_6_ binuclear cluster, is essential for fruiting body formation and virulence of *V. pyri*. The *VpFSTF1* deletion mutants exhibit similar growth phenotype as the WT on the normal medium, but are more sensitive to H_2_O_2_ and SA stresses in a dose dependent manner. VpFSTF1 may regulate expression of some virulence related genes, including well-known effector NPP1, catalase-related peroxidase and P450 superfamily. Finally, we demonstrated that VpFSTF1 could directly bind to the promoters of some genes, which encode the catalase-related peroxidase and P450 family protein. These results together reveal a role and possible mechanisms of VpFSTF1 in *V. pyri*, which may lay a foundation framework for further functional characterization of other fungal-specific TFs.

## Data Availability Statement

The datasets generated for this study can be found in the PRJNA588311.

## Author Contributions

FH and AK wrote the manuscript, and performed the experiments and data analysis. DD revised the manuscript and provided the funding for this research. AX revised the manuscript and designed experiments. JS, BL, XZ, and GA participated in manuscript revision or experiment.

## Conflict of Interest

The authors declare that the research was conducted in the absence of any commercial or financial relationships that could be construed as a potential conflict of interest.

## Footnotes

1 https://www.ncbi.nlm.nih.gov/blast

2 http://smart.embl-heidelberg.de

3 http://www.phi-base.org

## Supplementary Material

FIGURE S1 Generation of the *VpFSTF1* deletion mutant and complementation strains. **(A)** *ΔVpFSTF1*, deletion mutant, and complementation strain constructs were generated by PCR amplification (described in section “Materials and Methods”). The arrows indicate probe sites. The numbers (1–8) represent the primers (Supplementary Table S2) used for construction. **(B)** The mutant was confirmed by genomic PCR. WT, wild-type strain (Vp297); *ΔVpFSTF1* (K-29 and K-50), deletion mutant isolates obtained by PEG-mediated transformation; *VpFSTF1*com (C-140 and C-141), complementation strain isolates. **(C)** Transformant expression levels. Expression levels were quantified by qRT-PCR and normalized to actin gene expression. Expression levels of the mutant relative to those of WT are represented by fold changes calculated using 2^–ΔΔ*Ct*^.

FIGURE S2 Growth of the mutant on different carbon sources. **(A)** Colony growth on carbon sources. Mycelial agar plugs of wild type (WT), *ΔVpFSTF1* (K-29 and K-50), and *VpFSTF1*com (C-140 and C-141) were placed on agar media with different carbon sources, and the plates were incubated at 25°C in the dark for 24 h. Typical images of the colonies were taken at 24 h. **(B)** Radial growth of WT, *ΔVpFSTF1*, and *VpFSTF1*com on carbon sources. Colony diameters were measured at 24 h. **(C)** Fruiting bodies formed for WT and the complementation strain isolates (C-140 and C-141) but not for the K-29 and K-50 mutant isolates. Fruiting body formation was induced by growth under a cycle of 16 h light/8 h darkness for 15 days on PDA medium. The images were taken after 15 days.

FIGURE S3 RT–qPCR validations for DEGs. The twelve genes were using for RT-qPCR analyses. Columns indicate the relative expressions of genes were tested by the RT-qPCR, and linear graphs show the mean relative expressions data of genes that were predicted by RNA-seq. Relative expression of each gene was analyzed by RT-qPCR, normalized to actin expression levels and the relative expression ratio was calculated as the fold change (2^−ΔΔ*Ct*^) compared to the wild type (^∗^*p* < 0.05 or ^∗∗^*p* < 0.01, *t*-test). The relative expression levels of genes in RNA-seq were calculated using “WT, the mean RPKM (WT) value/the mean RPKM (WT) value or K-50: The mean RPKM (K-50) value/the mean RPKM (WT) value.” The mean relative expression levels of three replicates from RT-qPCR analyses were used for drawing graph. Each experiment was repeated at least three times.

TABLE S1 Transcript levels of several C6 transcription factor encoding genes.

TABLE S2 The primers used in this study.

TABLE S3 Differentially expressed genes (DEGs) controlled by VpFSTF1 involved in virulence.

TABLE S4 qRT-PCR validation of RNA-seq.
