# Supplementary figures and images for: The Fungal-Specific Transcription Factor VpFSTF1 Is Required for Virulence in Valsa pyri
Source: Front Microbiol. 2020 Jan 10;10:2945. doi: 10.3389/fmicb.2019.02945 (PMC6965324; doi:10.3389/fmicb.2019.02945)

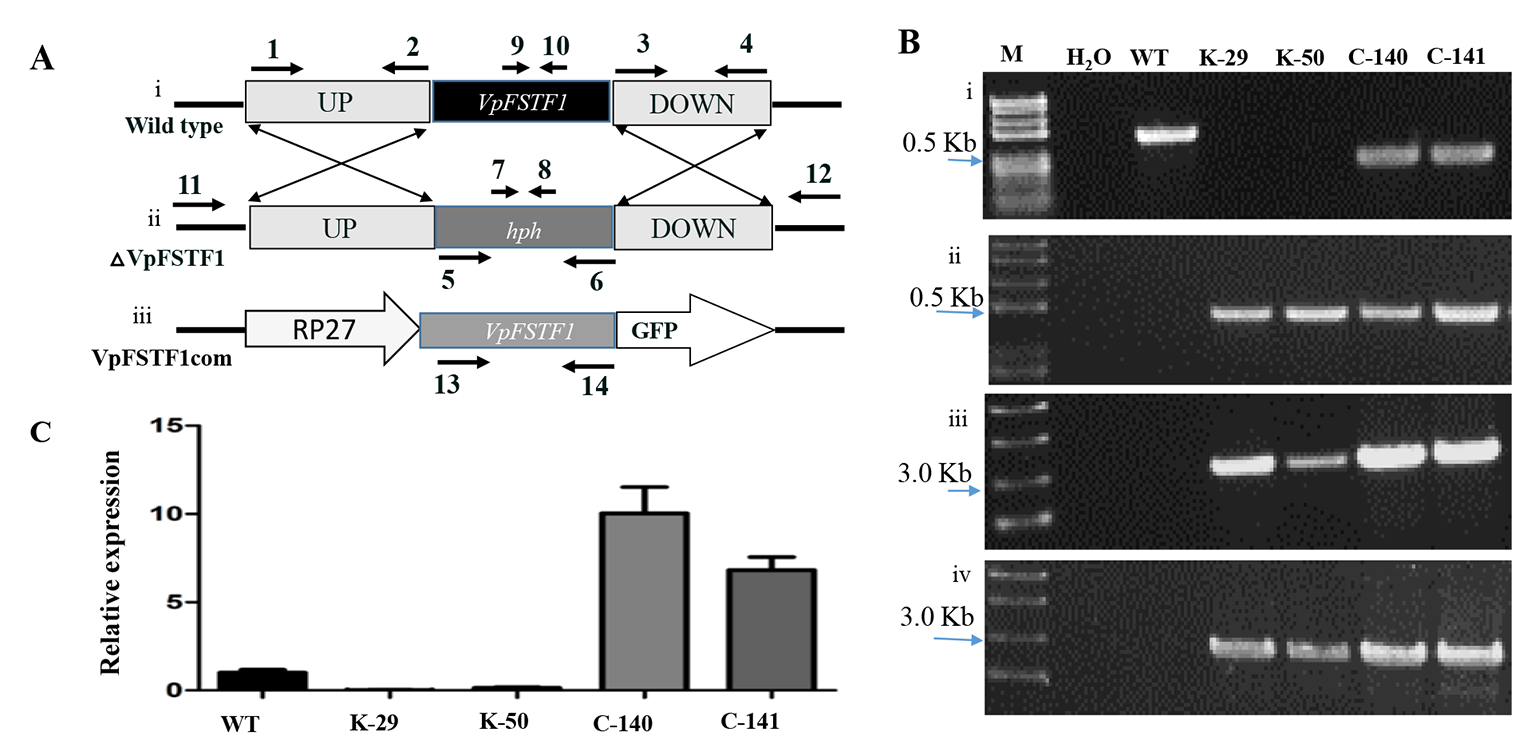

Supplement: FIGURE S1 — Generation of the VpFSTF1 deletion mutant and complementation strains. (A) ΔVpFSTF1, deletion mutant, and complementation strain constructs were generated by PCR amplification (described in section “Materials and Methods”). The arrows indicate probe sites. The numbers (1–8) represent the primers (Supplementary Table S2) used for construction. (B) The mutant was confirmed by genomic PCR. WT, wild-type strain (Vp297); ΔVpFSTF1 (K-29 and K-50), deletion mutant isolates obtained by PEG-mediated transformation; VpFSTF1com (C-140 and C-141), complementation strain isolates. (C) Transformant expression levels. Expression levels were quantified by qRT-PCR and normalized to actin gene expression. Expression levels of the mutant relative to those of WT are represented by fold changes calculated using 2–ΔΔCt. [file Image_1.tif]

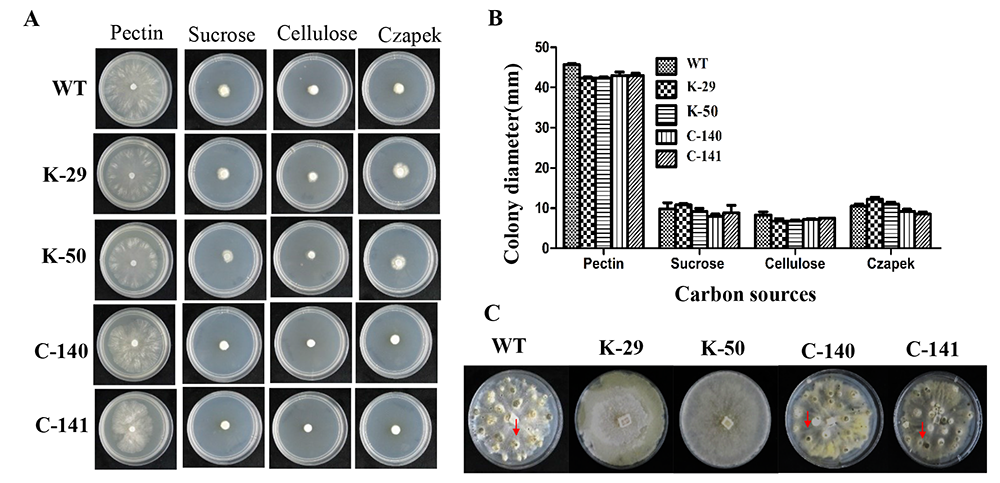

Supplement: FIGURE S2 — Growth of the mutant on different carbon sources. (A) Colony growth on carbon sources. Mycelial agar plugs of wild type (WT), ΔVpFSTF1 (K-29 and K-50), and VpFSTF1com (C-140 and C-141) were placed on agar media with different carbon sources, and the plates were incubated at 25°C in the dark for 24 h. Typical images of the colonies were taken at 24 h. (B) Radial growth of WT, ΔVpFSTF1, and VpFSTF1com on carbon sources. Colony diameters were measured at 24 h. (C) Fruiting bodies formed for WT and the complementation strain isolates (C-140 and C-141) but not for the K-29 and K-50 mutant isolates. Fruiting body formation was induced by growth under a cycle of 16 h light/8 h darkness for 15 days on PDA medium. The images were taken after 15 days. [file Image_2.tif]

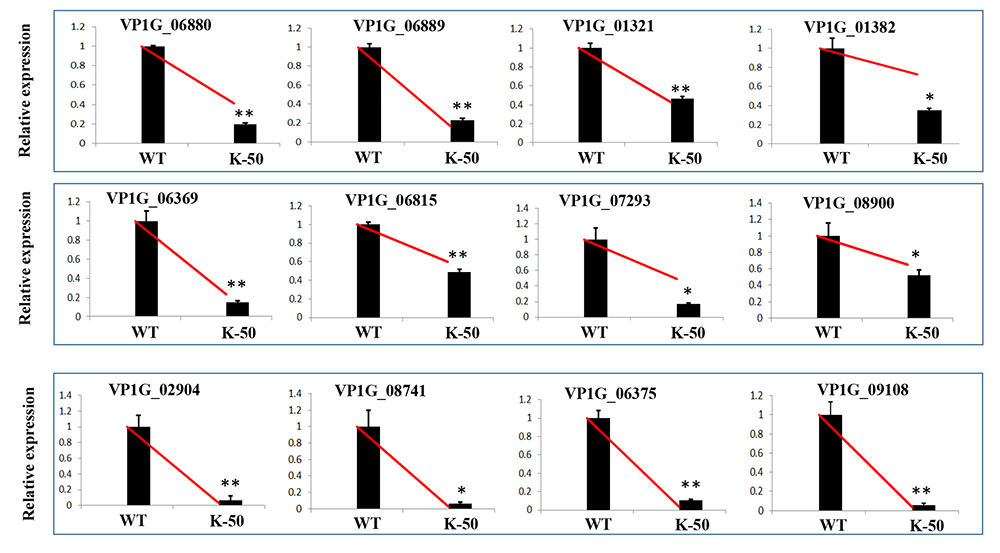

Supplement: FIGURE S3 — RT–qPCR validations for DEGs. The twelve genes were using for RT-qPCR analyses. Columns indicate the relative expressions of genes were tested by the RT-qPCR, and linear graphs show the mean relative expressions data of genes that were predicted by RNA-seq. Relative expression of each gene was analyzed by RT-qPCR, normalized to actin expression levels and the relative expression ratio was calculated as the fold change (2−ΔΔCt) compared to the wild type (∗p < 0.05 or ∗∗p < 0.01, t-test). The relative expression levels of genes in RNA-seq were calculated using “WT, the mean RPKM (WT) value/the mean RPKM (WT) value or K-50: The mean RPKM (K-50) value/the mean RPKM (WT) value.” The mean relative expression levels of three replicates from RT-qPCR analyses were used for drawing graph. Each experiment was repeated at least three times. [file Image_3.tif]
